# A Self-Refine-Enhanced LLM Framework for Structured Extraction of Quantitative Food Safety Information from Chinese Texts

**DOI:** 10.3390/foods15203700

**Published:** 2026-10-17

**Authors:** Zhiyao Zhao, Zhenzhen Zhao, Bojian Qi, Jiabin Yu

**Affiliations:** School of Computer and Artificial Intelligence, Beijing Technology and Business University, Beijing 100048, China

**Keywords:** food safety texts, large language models, triple extraction, Self-Refine, food safety supervision

## Abstract

Chinese food safety standards and scientific literature contain quantitative information relevant to food safety supervision and risk analysis. However, this information is dispersed across natural-language provisions and complex tables, making manual extraction labor-intensive and prone to omissions and incorrect associations. This study proposes a large language model (LLM)-based framework that combines few-shot prompting with Self-Refine to extract “food object–indicator/factor–numerical expression” triples. A corpus comprising 149 Chinese national food safety standards and 85 Chinese scientific articles was constructed, and the evaluation used 1031 reference triples from 27 documents. Across four runs, Qwen-Plus with Self-Refine achieved mean precision, recall, and F1 scores of 0.8690, 0.8041, and 0.8353 under strict matching and 0.8737, 0.8084, and 0.8398 under similarity-based matching. Strict-match precision and F1 increased by 0.0421 and 0.0207, respectively, while recall changed little. The F1 improvement remained positive across the tested similarity thresholds and field weights. Without additional model training, the proposed framework produces structured quantitative food safety information that can support digital archiving of food safety documents, information retrieval, and knowledge graph construction.

## 1. Introduction

With the growing complexity of global food supply chains, national food safety standards and supporting technical documents are continuously revised to incorporate more food varieties and hazard control requirements. These documents contain statutory limits for food additives, chemical contaminants, and pesticide residues, as well as microbiological criteria, test results, and risk assessment parameters. Food safety regulators and food producers use these provisions for regulatory sampling, internal compliance checks, and risk assessment [1,2]. However, the relevant information is embedded in unstructured text and complex tables across different document types and lacks a standardized representation. Manual extraction is time-consuming and may lead to omissions or incorrect associations because of hierarchical food classifications, complex table structures, and nested conditions. Therefore, extracting and associating the food object, indicator/factor, and numerical expression fields is necessary to structure information from heterogeneous food safety texts. The resulting quantitative food safety information can be organized, searched, and traced, and can serve as input for knowledge graph construction and risk analysis.

In recent years, natural language processing has been widely adopted for named entity recognition, relation extraction, and knowledge graph construction, laying a foundation for the automatic structuring of food safety texts [3,4]. Early approaches relied on rule templates, domain dictionaries or supervised learning, which demand heavy manual work for rule formulation and data annotation and show poor adaptability to diverse complex expressions in food safety standards. Pretrained language models and large language models (LLMs) have demonstrated capabilities in domain-specific text understanding, few-shot learning, and structured output generation. These capabilities provide an alternative approach to processing food safety documents when annotated data are limited and task-specific model training is not performed [5,6].

Existing studies have explored the structured processing and application of food safety texts. Chen et al. investigated Chinese named entity recognition on food safety standards to support entity boundary identification and category classification [7]. Subsequent research has advanced entity and relation extraction techniques for food safety regulations to facilitate knowledge graph development [8,9,10]. Yan et al. constructed a structured dataset and knowledge graph for forest food classification and nutritional information, providing a reference for structured knowledge organization in the food domain [11]. Relevant research also covers retrieval-augmented question answering for regulatory scenarios [12,13]. Recent advances in LLMs have brought new solutions for food safety text structuring. Recent studies have applied LLMs to chemical food safety hazard extraction and the classification of regulatory provisions, providing a basis for further investigation of structured information extraction from food safety texts [14,15].

These studies have established fundamental technical pathways for automated food safety text analysis, but their scope is largely confined to basic entity recognition, general relation extraction and text classification tasks. Few studies have targeted the core requirement of food safety supervision: extracting and associating complete elements, including food objects, indicators or factors, numerical expressions, and applicable constraints, in a unified manner. This gap makes existing research outputs difficult to be directly used as reliable data input for food safety supervision systems, and fails to support practical scenarios such as digital standard management, automated compliance checking and quantitative risk early warning, restricting the digital and intelligent transformation of food safety governance. To address this gap, this study defines the above multi-element associated regulatory information as quantitative food safety information. It covers limit specifications for food additives, contaminants and pesticide residues, as well as microbiological criteria, test data and risk assessment parameters documented in standards and the scientific literature, and is formalized as “food object–indicator/factor–numerical expression” triples to simultaneously record the applicable object, indicator or hazard factor, and numerical expression of each knowledge fact.

Quantitative food safety information is mostly embedded in conditional provisions and tables involving complex row–column correspondences. A numerical value may apply only to a specific food category, hazard factor, or qualifying condition, making food-object identification, indicator or hazard-factor assignment, and numerical alignment difficult. Although LLMs can generate candidate triples through single-pass extraction, their outputs frequently suffer from incorrect field associations, missing information and invalid records, reducing the reliability of structured data for regulatory purposes. The Self-Refine framework proposed by Madaan et al. iteratively improves model outputs through a closed loop of initial generation, self-feedback and refinement, enabling automatic inspection and field-level revision of extraction results [16]. This mechanism enables iterative review and revision of candidate triples during inference without updating model parameters.

Based on the above analysis, this study uses Chinese national food safety standards and scientific literature on food safety as its research corpus and proposes a quantitative food safety information extraction method combining structured few-shot prompting with Self-Refine-based iterative optimization. The main contributions are summarized as follows. First, a “food object–indicator/factor–numerical expression” triple extraction task is formulated. Tailored to the demands of food safety standards digitalization and regulatory information management, this unified representation scheme conforms to the data specifications of food safety risk database construction, and provides a standardized data carrier for limit provisions and risk assessment parameters. Second, a domain-specific corpus comprising 149 Chinese national food safety standards and 85 scientific articles on food safety is constructed. Tabular contents are linearized, and the resulting annotations are divided into a prompt example set and an independent evaluation reference set to support the extraction and objective evaluation of complex quantitative food safety information. Third, Self-Refine is introduced into quantitative food safety information extraction through initial extraction, feedback, inspection, and result revision. This method applies structured feedback and revision to improve the initial extraction results without additional model training. The proposed method delivers a standardized structured data basis for the digital archiving of food safety standards, knowledge graph construction and risk parameter management.

## 2. Materials and Methods

### 2.1. Data Construction

Existing public datasets in the food safety field are mainly designed for regulatory document retrieval, risk monitoring, food attribute analysis, or intelligent question answering. However, they do not provide a unified annotation scheme for quantitative constraint triples in food safety texts. Therefore, this study constructed a task-specific dataset based on national food safety standards and scientific literature. The data construction process included document collection, text preprocessing, and manual annotation.

A total of 234 documents were collected. The standard corpus consisted of 149 Chinese national food safety standards released from 1 January 2024 to the date of data collection and retrieved from the Foodmate.net standards and regulations database, whereas the literature corpus consisted of 85 Chinese scientific articles published within the past ten years and retrieved from China National Knowledge Infrastructure (CNKI) using food safety risk assessment as the main search topic. The national standards mainly provided normative information, such as food additive requirements, contaminant limits, pesticide residue limits, and microbiological criteria [17,18,19,20]. The scientific literature mainly provided research-oriented information, including detection results, risk factors, and risk assessment indicators. Together, these sources formed the experimental corpus and covered both normative and research-oriented food safety knowledge. The composition of the dataset, including the absolute number and percentage of documents from each source, is shown in Table 1.

During text preprocessing, all documents were converted into Markdown format and segmented into paragraph-level units. Empty and duplicated fragments were removed. Each retained paragraph was stored in JSON format together with its source information and paragraph identifier. For tabular contents in standards and scientific articles, tables were linearized while preserving headers, row–column relationships, values, and units. After preprocessing, a paragraph-level corpus with traceable source information was obtained.

An AI-assisted annotation procedure with manual verification was used to support prompt design and model evaluation. GPT-5.4 Pro generated preliminary annotations according to the predefined extraction schema. One of the authors conducted the initial manual verification and checked all candidate triples individually against the corresponding source texts. Incorrect triples were removed, and omitted triples were added based on the source texts to produce the final annotations. The resulting annotated data were divided into two non-overlapping subsets: a prompt example set and an evaluation reference set. The prompt example set was used only to construct few-shot prompts, whereas the evaluation reference set was used only as the benchmark for model evaluation. The two subsets did not overlap at either the document level or the paragraph level, thereby reducing the risk of data leakage.

After excluding texts used in the prompt example set, candidate documents were selected at the document level and manually reviewed according to the triple extraction task. Documents containing food objects, indicators or factors, numerical constraints, detection results, or risk assessment parameters were retained, whereas background-only or concept-explanation texts without valid triples were excluded. The final evaluation reference set contained 27 documents, including 5 national standards and 22 scientific articles. Only a limited number of the retrieved national standards contained explicit numerical safety limits that met the inclusion criteria. The sample ratio of literature to regulations was not pre-specified, but emerged after full retrieval and uniform manual quality screening of all available candidates. The imbalance between sources is noted as a limitation of this study.

For the selected evaluation reference set, annotations of valid sentences or paragraphs followed the AI-assisted annotation and manual verification procedure described above, using three core fields: subject, predicate, and object. Specifically, subject records the food object, predicate records the corresponding indicator, factor, or detection item, and object records the numerical expression associated with the predicate. Auxiliary fields, including unit, constraint_symbol, source_type, knowledge_type, and relation_type, were used for normalization, source tracing, and error analysis. In the subsequent matching-based evaluation, subject, predicate, and object were used as the main matching fields.

To preserve the applicable scope of quantitative regulatory facts, the final output may include a conditions array in addition to the subject–predicate–object fields. This field records directly supported qualifications, explicit applicability exceptions, and parent-category–subcategory relationships explicitly represented in source tables. Each condition is associated with the corresponding numerical assertion and is retained only when it has direct textual support and can be uniquely linked to that assertion. For tabular content, hierarchical information is completed only when the parent–child relationship is explicit in the original table; otherwise, no new hierarchy is inferred.

Written annotation guidelines were used to define the extraction scope and the “food object–indicator/factor–numerical expression” triple structure. The guidelines specified the boundary rules for food objects and indicators/factors; the retention of numerical values, constraint symbols, ranges, units, and applicable conditions; and the interpretation of table headers and row–column relationships. Statements containing only detection methods or references to other standards without explicit quantitative information were excluded. Duplicate triples were removed, while missing or uncertain triples were re-examined against the corresponding source texts.

To assess annotation reliability, a second graduate researcher in artificial intelligence independently audited a subset comprising eight documents, including four national food safety standards and four scientific articles, with a total of 60 source-text units. Each unit was either a natural-language paragraph or a complete table with its structure preserved. The second annotator had experience in information extraction and structured annotation of Chinese texts, had not participated in the initial annotation verification, and reviewed the written guidelines and worked examples before the audit. The annotator independently examined the selected source-text units and produced a separate set of records before comparison with the original annotations. Before adjudication, the original annotations contained 509 triples and the independent audit contained 577 triples, of which 475 were strictly matched across the food object, indicator/factor, and numerical expression fields. Against the original annotations, the independent audit achieved a precision of 82.32% (475/577), recall of 93.32% (475/509), and F1 score of 87.48%. The unmatched records were subsequently re-examined against the source texts and resolved according to the annotation guidelines.

After field-completeness checking and format standardization, the evaluation reference set contained 1042 annotation records. Of these, 1031 records had complete subject, predicate, and object fields and were therefore treated as valid reference triples in the evaluation. The field composition and annotation scale of the evaluation reference set are summarized in Table 2.

According to the source information, 820 valid triples were derived from national standards and 211 from scientific literature. Overall, the constructed dataset covered the main types of quantitative food safety information and provided a unified data basis for subsequent prompt-based extraction, Self-Refine optimization, and matching-based evaluation.

### 2.2. Overall Methodological Framework

**Figure 1:**
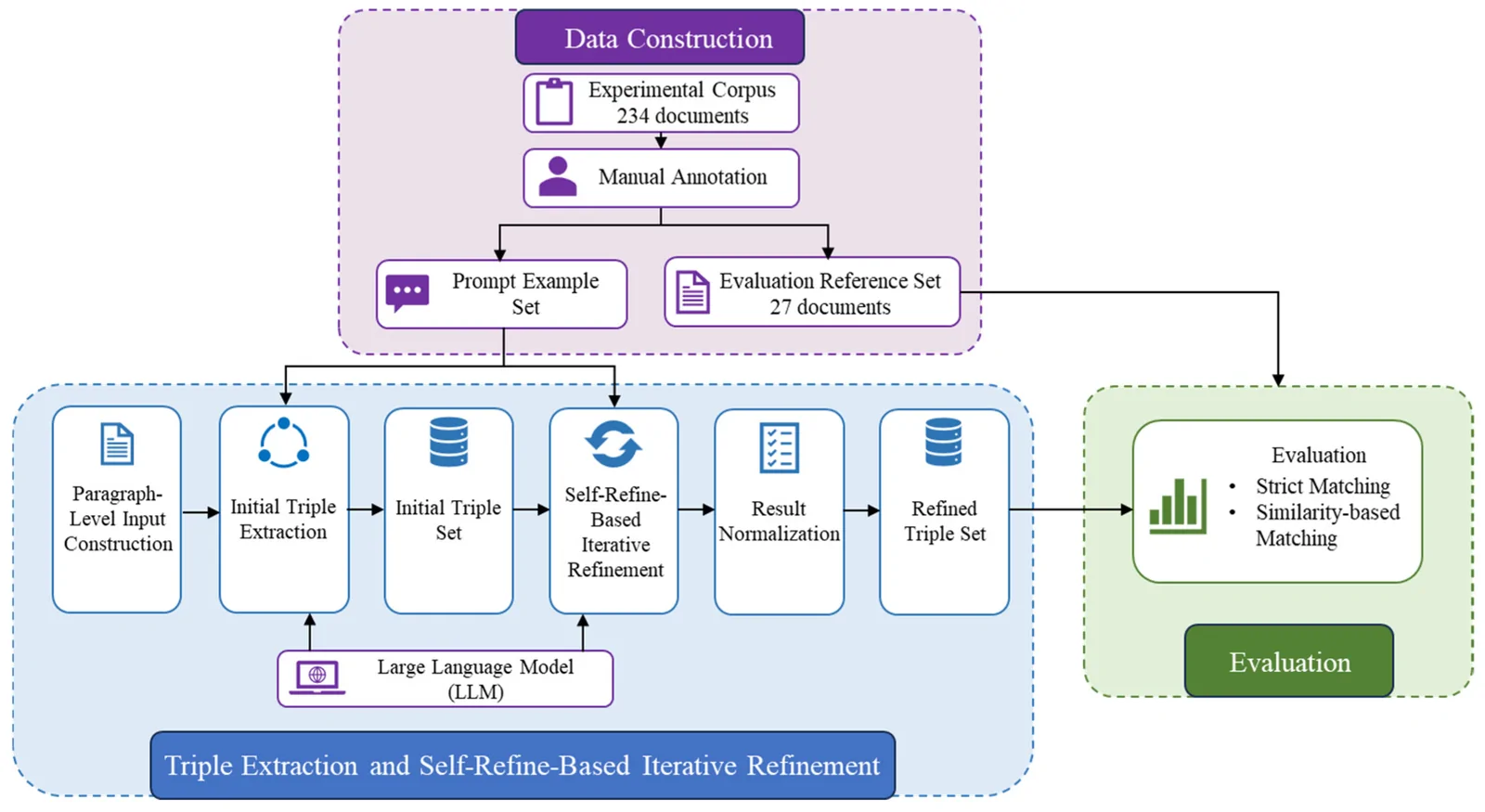
Overall framework for extracting quantitative food safety information from food safety texts.

Based on the data construction described in Section 2.1, this study proposes a framework for extracting and refining quantitative food safety information from food safety texts. The framework consists of three modules: Data Construction, Triple Extraction and Self-Refine-Based Iterative Refinement, and Evaluation, forming a complete workflow from data preparation to performance evaluation, as shown in Figure 1.

The Data Construction module prepares the experimental corpus and reference annotations. The collected documents are preprocessed into a paragraph-level corpus, including the linearization of tabular contents. The annotated data are then divided into a prompt example set for few-shot prompting and an evaluation reference set that is disjoint from the prompt example set.

The Triple Extraction and Self-Refine-Based Iterative Refinement module uses preprocessed paragraphs as the basic input units. Whole-document or long-section inputs may introduce interference among unrelated clauses, whereas sentence-level inputs may omit essential contextual information, particularly table headers, applicable conditions, and row–column relationships in linearized tables. Paragraph-level input was therefore adopted to balance contextual completeness and extraction focus. However, single-pass LLM extraction may still produce inaccurate, incomplete, or redundant triples. Considering that parameter fine-tuning usually requires additional training and computational resources, this study introduces a Self-Refine strategy without parameter updates to improve extraction accuracy and standardization. The LLM first generates an initial triple set, which is then reviewed and revised according to the original text and structured feedback. Finally, result normalization standardizes field expressions and removes duplicate triples to obtain the refined triple set.

The Evaluation module compares the refined triple set with the reference triples in the evaluation set. Strict matching and similarity-based matching are applied to the subject, predicate, and object fields, and precision, recall, and F1 score are calculated to evaluate triple extraction performance.

### 2.3. Prompt Design and Initial Triple Extraction

**Figure 2:**
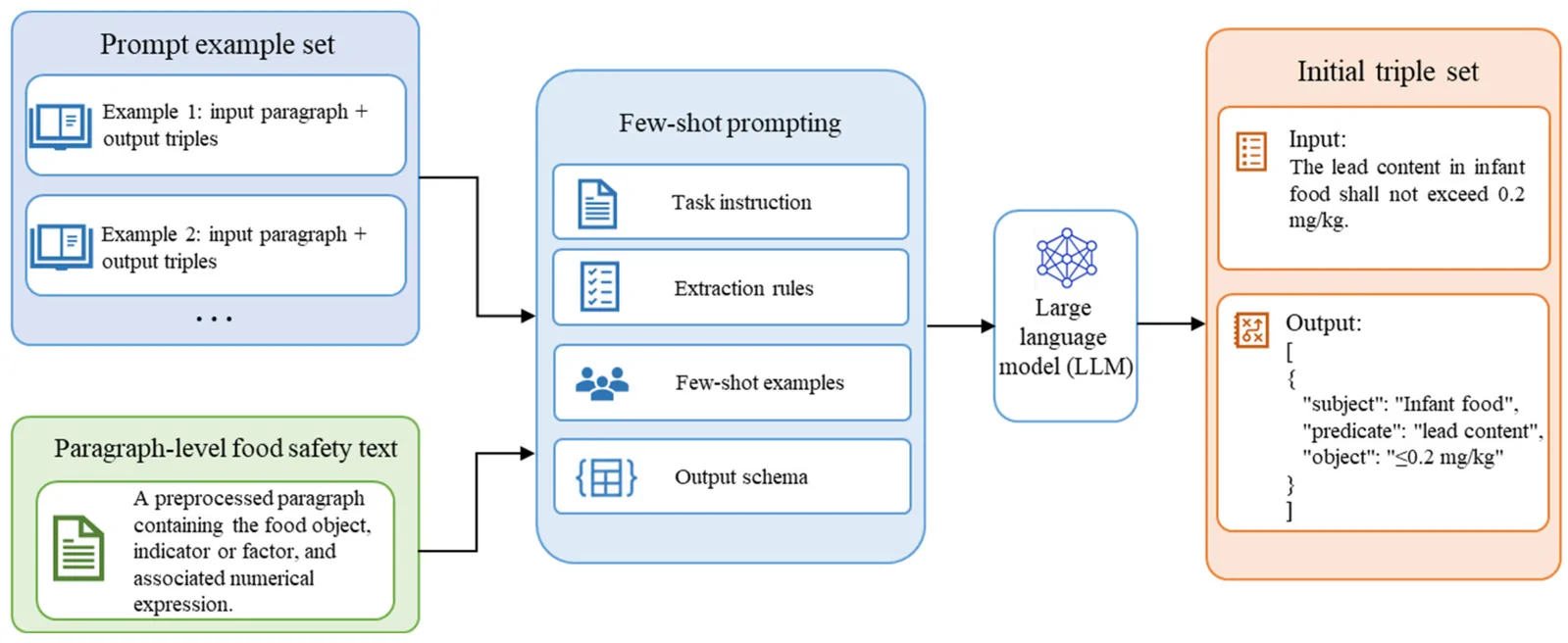
Workflow of paragraph-level triple extraction driven by few-shot prompting.

Since no parameter fine-tuning was performed in this study, initial triple extraction mainly relied on prompts to guide the LLM’s task understanding and output behavior. To generate structurally consistent and parsable candidate triples from paragraph-level food safety texts, few-shot prompting was combined with prompt engineering. The overall process includes paragraph-level text input, prompt construction, LLM-based candidate triple generation, result parsing and preliminary processing, and initial triple set construction. The workflow of paragraph-level initial triple extraction driven by few-shot prompting is shown in Figure 2.

Few-shot prompting was used to provide representative extraction demonstrations. Several manually checked examples were embedded in the prompt as in-context demonstrations. These examples covered common expression forms in food safety texts, including natural-language clauses, numerical limit requirements, range expressions, unit expressions, and linearized table contents. Each example consisted of an input paragraph and its corresponding structured output, enabling the model to learn the expected mapping from paragraph-level text to structured triples [21].

Prompt engineering was used to constrain the extraction behavior of the LLM. The initial extraction prompt consisted of four components: task instruction, extraction rules, few-shot examples, and output schema. The task instruction specified the extraction objective. The extraction rules required the model to retain only triples directly supported by the input text and to process natural-language clauses and linearized tables according to their respective structures. The output schema required the model to return structured records following the predefined field format described in Section 2.1, facilitating subsequent parsing, normalization, and evaluation.

During initial extraction, each preprocessed paragraph was independently inserted into the prompt template. The LLM generated candidate triples according to the task instruction, extraction rules, few-shot examples, and output schema. The generated outputs were then parsed into structured records. Empty outputs, incorrectly formatted records, and duplicate triples within the same paragraph were removed during preliminary processing. The remaining candidate triples formed the initial triple set, which was subsequently used as the input to the Self-Refine-based iterative refinement process.

**Figure 3:**
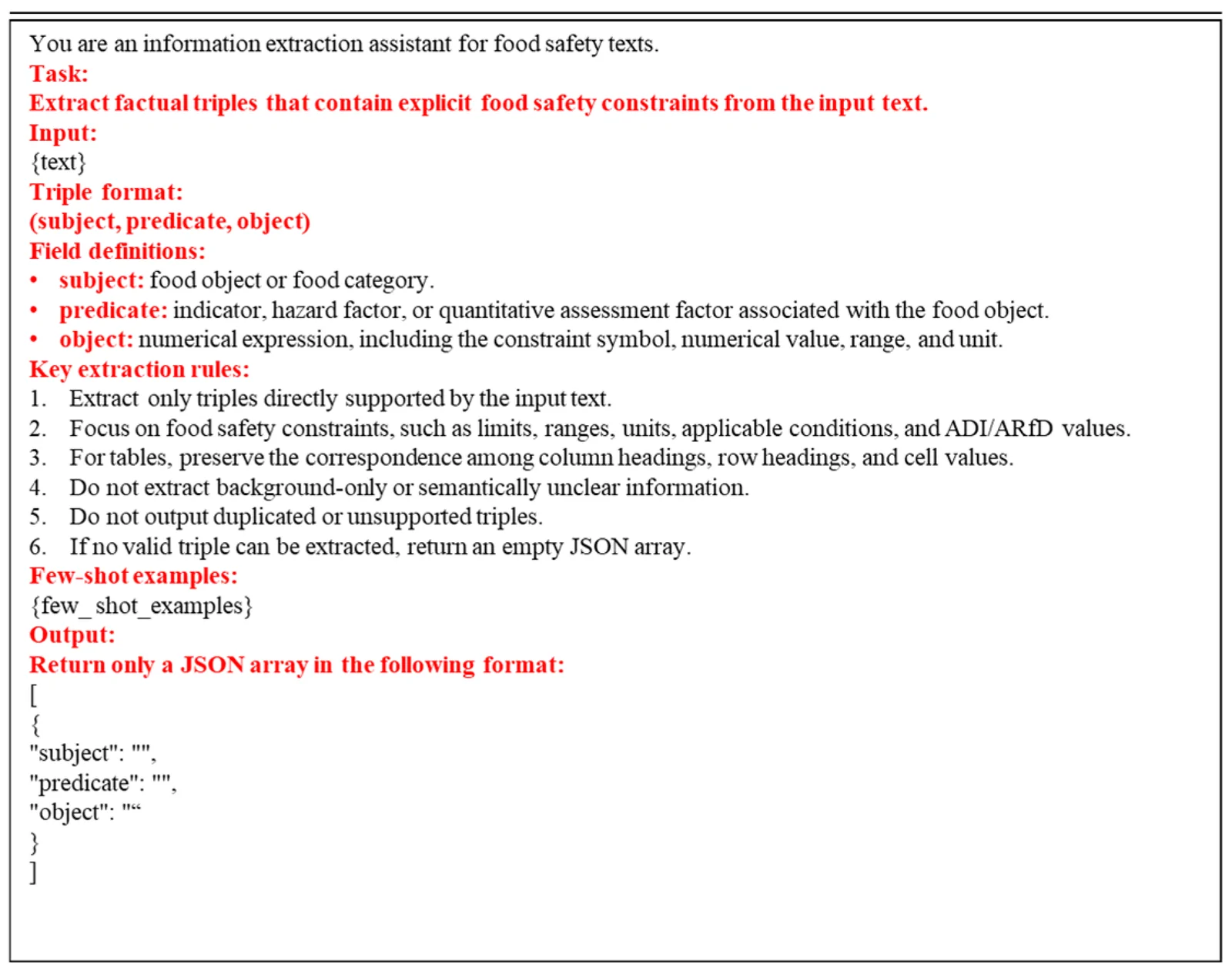
Simplified prompt template for initial triple extraction.

The simplified prompt template for initial triple extraction is shown in Figure 3.

### 2.4. Self-Refine-Based Iterative Refinement

Since the initial extraction results may still contain inaccurate field boundaries, incorrect indicator assignment, missing value–unit information, and duplicated or unsupported triples, this study further introduced a Self-Refine-based iterative refinement mechanism to revise the initial triple set obtained in Section 2.3. Following the Self-Refine framework proposed by Madaan et al. [16], the generation–feedback–revision process was adapted to the food safety text triple extraction task. This mechanism did not update the parameters of the LLM. Instead, the same LLM was used during inference to review the current triple set, generate structured feedback, and revise the extraction results.

For a paragraph-level input x, the initial triple set is denoted as $T_{0}$. At iteration t, the LLM first generates structured feedback $F_{t}$ based on the original text x and the current triple set $T_{t}$. The feedback focuses on text support, food-object boundaries, indicator assignment, numerical expressions, units, and duplicated or missing triples. The LLM then produces a revised triple set $T_{t+1}$ according to the feedback. After the stopping condition is satisfied, normalization is applied to obtain the final refined triple set $T^{*}$.

Two prompts were designed for this process: a feedback prompt for generating structured error information and a refinement prompt for revising candidate triples according to the feedback. The complete Self-Refine-based iterative refinement process is summarized in Algorithm 1.
**Algorithm 1** Self-Refine-based iterative refinement for food safety triple extraction**Require:** paragraph-level text *x*, initial triple set *T*_0_, maximum number of iterations *T*_max_ = 3 **Output:** refined triple set $T^{*}$1: Set t = 0.2: **while** t < $T_{\max}$ **do**
3:      Generate structured feedback $F_{t}$ for $T_{t}$ based on x.4:      if $I_{t}=\emptyset ,\;M_{t}=\emptyset$, and $R_{t}=\emptyset$ then.5:            break.6:      **end if**7:      Generate the revised triple set $T_{t+1}$ using $F_{t}$.8:      if $N\left(T_{t+1}\right)=N\left(T_{t}\right)$ then9:            Set $T_{t}=T_{t+1}$.10:          break.11:    **end if**12:    Set $T_{t}=T_{t+1}$.13:    Set t=t+1.14: **end while**15: Set $T^{*}=N\left(T_{t}\right)$ and perform format validation.16: return $T^{*}$.

Here, $I_{t}$, $M_{t}$, and $R_{t}$ denote the invalid_triples, missing_triples, and revision_suggestions lists in the feedback, respectively, and N(⋅) denotes triple normalization and duplicate removal.

**Figure 4:**
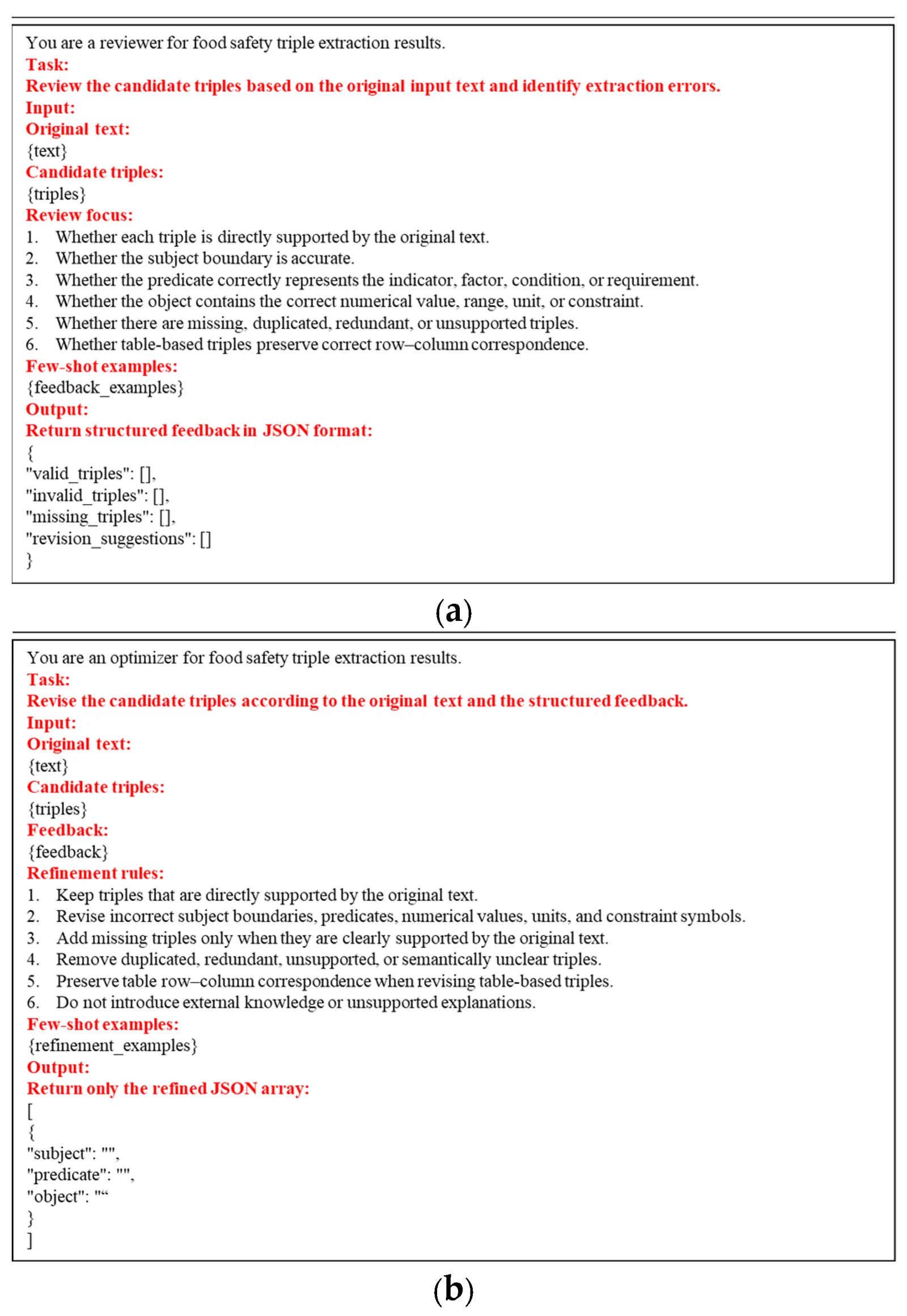
Simplified prompt templates for Self-Refine-based refinement. (**a**) Feedback prompt template; (**b**) refinement prompt template.

The simplified templates of the feedback prompt and refinement prompt are shown in Figure 4.

During iterative refinement, unsupported or erroneous triples were removed or corrected, missing triples were added only when explicitly supported by the original paragraph, and inconsistent field expressions were standardized. After the stopping condition was satisfied, the resulting triple set was normalized to obtain the final extraction result for each paragraph. Structured feedback and constrained revision were jointly used to improve the accuracy and standardization of quantitative food safety information extraction. From a practical perspective, food-category misclassification, incorrect limit values, and unit confusion can reduce the reliability of structured food safety standards data. The feedback–revision process was designed to check the food object, indicator or factor, numerical value, unit, and constraint symbol against the original paragraph and revise identified inconsistencies.

### 2.5. Evaluation Metrics

Before evaluation, model outputs were normalized by removing empty or duplicated triples, standardizing units and constraint symbols, and normalizing selected indicator aliases. The processed triples were then matched with the reference triples based on subject, predicate, and object.

Two matching strategies were used: strict matching and similarity-based matching. Strict matching required the normalized subject, predicate, and object fields of a predicted triple to be identical to those of a reference triple. Strict matching was treated as the primary evaluation criterion, whereas similarity-based matching provided a complementary assessment allowing minor differences in field wording.

Similarity-based matching followed a string-based approach to approximate matching [22]. After character cleaning, whitespace removal, and symbol normalization, subject and predicate similarities were calculated using SequenceMatcher from the Python 3.12.7 difflib module. This function measures agreement between character sequences and returns a similarity score between 0 and 1. The numerical object was compared in terms of its constraint symbol, numerical value or range, and unit. When the constraint symbol or numerical value was inconsistent, the object similarity was set to 0. Units were compared after normalization.

For reference targets containing a necessary qualification or exception, condition-aware correctness was additionally checked using the final persisted output. Such a target was judged correct only when the required condition appeared in the subject, predicate, or object, or in a conditions entry explicitly associated with the corresponding assertion. Feedback text, intermediate outputs, and conditions that were not included in the final output were not used for evaluation. If conflicting numerical values for the same target remained in the final output, the prediction was judged incorrect even when its conditions were complete.

The overall similarity score was calculated as follows:

(1) $$Score=w_{s}S_{s}+w_{p}S_{p}+w_{o}S_{o}$$

where $S_{s}$, $S_{p}$, and $S_{o}$ denote the similarities of the subject, predicate, and object fields, respectively, and $w_{s}$, $w_{p}$, and $w_{o}$ denote their corresponding weights.

Based on the matching results obtained under each strategy, *precision*, *recall*, and *F*1 score were calculated separately to evaluate triple extraction performance. *Precision* represents the proportion of predicted triples that match the reference triples, *recall* represents the proportion of reference triples recovered by the method, and the *F*1 score is the harmonic mean of *precision* and *recall* [23]:

(2) $$Precision=\frac{TP}{TP+FP}$$

(3) $$Recall=\frac{TP}{TP+FN}$$

(4) $$F1=\frac{2\times Precision\times Recall}{Precision+Recall}$$

where *TP* denotes correctly matched triples, *FP* denotes extracted triples not matched with the reference triples, and *FN* denotes reference triples not correctly extracted by the model.

For the original similarity-based evaluation configuration, the subject, predicate, and object weights were set to ($w_{s}$ = 0.35), ($w_{p}$ = 0.25), and ($w_{o}$ = 0.40), respectively. The object received the largest weight because its numerical value, constraint symbol, and unit determine the quantitative meaning of an extracted fact. The weighting approach was informed by previous attribute-normalization research [24], while the specific values reflected the relative importance assigned to the three fields. A predicted–reference triple pair was eligible for matching when its overall similarity was at least 0.85 and its predicate similarity was at least 0.75. Eligible pairs were assigned using a deterministic greedy one-to-one procedure within the same document and paragraph. Reference triples were processed in their stored order after duplicate removal. For each reference triple, the evaluator selected the highest-scoring eligible prediction from those not previously assigned and immediately marked both triples as used. Previously assigned pairs were not reconsidered. If multiple eligible predictions had the same highest score, the prediction appearing first in the stored prediction list was selected; no random tie-breaking, backtracking, or global reassignment was applied.

The original similarity-based matching configuration was fixed and applied consistently across all model comparisons and Self-Refine evaluations. A sensitivity analysis was conducted to examine whether the observed Self-Refine improvement remained consistent across alternative overall thresholds and field-weight configurations.

## 3. Experiments and Results

### 3.1. Experimental Settings and Evaluation Methods

The experiments comprised four main components: base-model selection, comparison with a non-LLM rule-based baseline, Self-Refine ablation, and sensitivity analysis of similarity-based matching. In the base-model selection experiment, Qwen-Plus (version: qwen-plus-2025-12-01), DeepSeek-V4-Pro, and Kimi-k2.5 were compared using precision, recall, and F1. The base model for subsequent Self-Refine experiments was selected according to its mean F1 performance under strict and similarity-based matching. The rule-based baseline provided a conventional non-LLM reference for initial extraction. Using the selected base model, the Self-Refine ablation compared initial and refined outputs to evaluate the additional effect of feedback-based revision.

Qwen-Plus, DeepSeek-V4-Pro, and Kimi-k2.5 were selected as candidate models based on their Chinese-language processing capabilities, task-relevant features, and availability through official APIs. Specifically, Qwen-Plus supports structured output generation, DeepSeek-V4-Pro offers language understanding and reasoning capabilities, and Kimi-k2.5 supports language understanding and contextual information processing. These capabilities motivated their inclusion in the comparative evaluation of food object–indicator/factor–numerical expression triple extraction, with their task-specific performance assessed experimentally. All three models were accessed through their respective providers’ official APIs on 13 May 2026. Qwen-Plus was accessed using the model identifier qwen-plus, corresponding to the snapshot version qwen-plus-2025-12-01.

Model selection and subsequent evaluations used the same reference set described in Section 2.1, with the prompt example set excluded from evaluation. All methods used the same preprocessed texts, output normalization procedures, and evaluation criteria. The three LLMs used the same initial extraction prompt and output schema. Four few-shot examples were used for initial extraction, while three examples were used for feedback generation and three for refinement. This configuration provided demonstrations of the main expression patterns and typical error types while limiting prompt length. For initial extraction, the temperature was set to 0.2, with maximum output lengths of 800 tokens for text paragraphs and 12,000 tokens for linearized tables per API call. For both the feedback and refinement stages of Self-Refine, the temperature was set to 0.1, and the maximum output length was 4000 tokens per API call. The top_p and seed parameters were handled according to each provider’s defaults at the time of access. Following the limited-iteration design of the original Self-Refine framework [16], $T_{\max}$ = 3 was adopted as a practical upper bound to allow multiple feedback–refinement rounds while controlling additional inference overhead. Refinement could terminate earlier when the feedback indicated that no further revision was required or when the normalized triple set remained unchanged. To provide additional empirical support for this setting, a small-scale exploratory analysis was conducted on a paragraph subset, comparing maximum refinement limits from zero to four rounds under strict matching. Three rounds yielded the highest observed F1 among the tested settings, while increasing the limit to four rounds provided no further improvement. These results support the use of three rounds as a practical upper bound in the present framework.

The deterministic rule-based baseline combined domain-lexicon matching, regular-expression-based numerical recognition, and field-association rules. Food objects and indicators or factors were identified using predefined domain lexicons, whereas numerical values, ranges, units, and constraint symbols were recognized using regular expressions. Candidate fields within the same sentence or linearized table row were associated according to textual proximity and structural position. When multiple candidates occurred, the nearest compatible fields were paired. Records lacking any of the three core fields were removed. The lexicons and rules were fixed before evaluation and were not subsequently modified according to the evaluation results.

Considering the stochasticity of LLM-generated outputs, each setting in the base-model comparison and the main Self-Refine ablation was independently repeated four times with unchanged prompts, inference parameters, and output schemas for the corresponding stages. Precision, recall, and F1 were calculated separately for each run and summarized as mean ± standard deviation (SD). The deterministic rule-based baseline was evaluated once. For the Self-Refine ablation, initial and refined results from the same run were paired. Differences were calculated from unrounded per-run metrics as the value with Self-Refine minus the value without Self-Refine. Approximate 95% confidence intervals (CIs) for the mean differences were calculated from the four paired differences using the t distribution with three degrees of freedom. These intervals describe variation across runs on the fixed evaluation set, rather than uncertainty associated with sampling different documents, and should be interpreted in the context of the four repeated runs.

For sensitivity analysis, the overall similarity threshold was varied from 0.75 to 0.95 in increments of 0.05. The original field weights ($w_{s}$ = 0.35, $w_{p}$ = 0.25, $w_{o}$ = 0.40) were compared with equal weights ($w_{s}=w_{p}=w_{o}=1/3$), while the minimum predicate-similarity threshold remained fixed at 0.75. Retained model outputs were re-evaluated under each configuration without additional LLM inference. All other normalization, numerical-object comparison, and one-to-one matching rules remained unchanged.

Two focused supplementary analyses were conducted to examine repeated extraction and aggregation strategies and model responses to perturbed or target-free inputs.

First, to compare repeated extraction and aggregation strategies, an exploratory analysis was conducted on a document-balanced subset of 40 source-text units, each comprising either a natural-language paragraph or a linearized table. Three settings were evaluated on this subset: single-pass extraction, majority voting over four independent initial-extraction outputs, and Self-Refine with up to three refinement rounds. The voting rule retained triples appearing in at least three of the four outputs. The subset-selection procedure and further experimental details are provided in Appendix A (Appendix A.1), and the complete performance results are reported in Table A1. Inference resource use was recorded as the number of API calls, the number of output tokens returned by the API, and aggregate API response time. The aggregate response time was defined as the sum of the response times of the individual requests, and the reported token counts include output tokens only.

Second, an exploratory diagnostic assessment was conducted using 40 original positive passages, 40 corresponding passages with controlled text perturbations, and 30 negative passages, totaling 110 input passages. Each original positive passage had one corresponding perturbed version. The assessment examined target accuracy on the original positive inputs, correct perturbation following on the perturbed inputs, and the non-empty output rate on the negative inputs. Target accuracy was defined as the proportion of original positive passages for which the expected target triple was correctly extracted. A perturbed passage was counted as correctly followed only when the extracted target triple matched the expected triple after perturbation, including the deliberately modified field, rather than retaining the corresponding value from the original passage.

The negative set was constructed through purposive selection with category quotas from existing food safety standards and scientific literature. It comprised 30 original passages from four standards and eight scientific articles, contributing 11 and 19 passages, respectively. Three categories were included, with 10 passages per category: background or conceptual descriptions, passages containing only references to other standards, and passages containing non-target numerical distractors. Selection was based on the absence of an eligible food object–indicator/factor–numerical expression relationship within the supplied passage. Years, standard identifiers, clause numbers, and sampling-batch identifiers were distinguished from numerical expressions belonging to target relationships. Each passage retained its traceable source location and necessary headings. The negative set was confirmed and fixed before model execution, and no passages were replaced or excluded based on model outputs. The expected output for each negative passage was an empty triple set. The non-empty output rate was calculated as the number of negative passages yielding at least one extracted triple divided by the total number of negative passages.

After the final model and experimental configuration were fixed, an independent test set of 10 previously unused documents was evaluated, comprising four national food safety standards and six scientific articles with 481 valid reference triples. These documents were excluded from all development, design, model and parameter selection, prompt construction, and main evaluation-set construction stages, with non-overlap verified using document identifiers and text content. Initial extraction and Self-Refine were evaluated using the unchanged experimental configuration, with micro-averaged metrics calculated across all 10 documents. Supplementary analyses of the Self-Refine outputs included stratification by document source and content format and a document-level stratified bootstrap analysis. Both analyses were conducted under strict matching and are reported in Appendix A.2 and Appendix A.3, respectively.

### 3.2. Base-Model Selection and Comparison with a Rule-Based Baseline

To select a base model for the subsequent Self-Refine experiment, the initial triple extraction performance of Qwen-Plus, DeepSeek-V4-Pro, and Kimi-k2.5 was compared. A deterministic rule-based baseline was also included to provide a conventional non-LLM reference. The comparison followed the experimental settings described in Section 3.1.

The three LLMs were accessed through API services. The local environment was used for text preprocessing, prompt construction, API request scheduling, output parsing, result normalization, and metric calculation. The initial extraction results under strict and similarity-based matching are presented in Table 3.

As shown in Table 3, Qwen-Plus achieved the highest mean F1 among the three LLMs under both strict matching and similarity-based matching, with scores of 0.8146 and 0.8190, respectively. DeepSeek-V4-Pro obtained the highest recall but lower precision than Qwen-Plus, indicating that its greater coverage of reference triples was accompanied by a larger proportion of unmatched predictions. Kimi-k2.5 had the lowest precision, recall, and F1 among the three LLMs under both matching criteria.

The rule-based baseline achieved F1 scores of 0.5360 under strict matching and 0.7863 under similarity-based matching. Under similarity-based matching, it obtained the highest precision among the compared methods, reaching 0.8809. However, its recall was lower than that of Qwen-Plus (0.7100 versus 0.8070), resulting in a lower F1 score. Thus, the rule-based method produced a higher proportion of matched predictions under the complementary criterion while recovering fewer reference triples. The substantial difference between its strict and similarity-based F1 scores also indicates that its measured performance was strongly affected by relaxing exact field agreement.

Overall, Qwen-Plus achieved the highest mean F1 among the three LLMs and higher F1 than the rule-based baseline under both matching criteria. Based on this balance between precision and recall, Qwen-Plus was selected as the base model for the Self-Refine ablation experiment.

### 3.3. Self-Refine Ablation and Supplementary Analyses

#### 3.3.1. Ablation Results and Supplementary Comparisons

To evaluate the contribution of feedback-based revision, the Qwen-Plus outputs before and after Self-Refine were compared under the experimental settings described in Section 3.1. Table 4 presents the ablation results and run-level paired differences.

As shown in Table 4, Self-Refine increased mean precision by 0.0421 under strict matching and 0.0423 under similarity-based matching, with corresponding F1 increases of 0.0207 and 0.0208. Precision and F1 improved in all four paired runs, and their run-level 95% CIs excluded zero under both matching criteria. In contrast, the mean paired increase in recall was approximately 0.0015 under both criteria, and both recall CIs included zero. The four-run analysis therefore supported consistent improvements in precision and F1 but did not provide clear evidence of a consistent recall improvement, suggesting that Self-Refine mainly increased the proportion of correctly matched predictions rather than substantially expanding coverage of the reference triples.

Comparing Table 3 and Table 4, Qwen-Plus with Self-Refine achieved higher F1 than the rule-based baseline under strict matching (0.8353 versus 0.5360) and similarity-based matching (0.8398 versus 0.7863). The rule-based baseline achieved higher precision under similarity-based matching (0.8809 versus 0.8737), whereas the refined Qwen-Plus outputs had higher recall (0.8084 versus 0.7100). Thus, the refined LLM outputs provided a better overall balance between precision and recall on the evaluated reference set.

To examine whether repeated extraction followed by majority voting could achieve improvements comparable to Self-Refine, we conducted an exploratory comparison on the 40-unit subset described in Appendix A.1. Under strict matching, the F1 scores of single-pass extraction, majority voting over four independent outputs, and Self-Refine were 85.20%, 84.45%, and 86.61%, respectively. Under similarity-based matching, the corresponding F1 scores were 90.82%, 89.51%, and 91.03%. The complete precision, recall, and F1 results are reported in Table A1.

For inference-resource accounting, single-pass extraction, majority voting, and Self-Refine required 40, 160, and 138 API calls, respectively; returned 98,628, 394,451, and 470,599 output tokens; and had aggregate API response times of 166.4, 661.9, and 1406.3 s. Their returned output-token use was therefore 1.00×, 4.00×, and 4.77× that of single-pass extraction, respectively. The actual number of Self-Refine calls was below the theoretical maximum because refinement could terminate before reaching three rounds.

On this small subset, Self-Refine achieved the highest F1 under both strict and similarity-based matching, whereas majority voting did not improve F1 over single-pass extraction. The results provide supplementary evidence for the contribution of feedback-guided revision while also showing the associated inference-resource requirements. Given the limited subset size, this comparison is interpreted as an exploratory analysis.

#### 3.3.2. Independent Validation and Diagnostic Assessment

To assess whether the improvement observed on the main evaluation set also occurred on previously unused documents, the fixed framework was evaluated on the independent test set described in Section 3.1. As shown in Table 5, Self-Refine increased strict-match F1 from 76.46% to 81.05% and similarity-based F1 from 82.42% to 86.10%, with improvements in both precision and recall under both criteria. The direction of the F1 improvement was consistent with the main ablation results, providing additional evidence from the independent test set.

To examine variation across extraction settings and uncertainty associated with document sampling, supplementary strict-match analyses were conducted for the Self-Refine outputs. Results stratified by document source and content format are presented in Appendix A.2 (Table A2 and Table A3), and document-level bootstrap confidence intervals are reported in Appendix A.3 (Table A4).

In addition to evaluating extraction performance on the independent test set, a focused diagnostic assessment was conducted to examine whether Self-Refine followed controlled changes in the target information and reduced unsupported extraction from passages without eligible targets. On the 40 original positive passages, target accuracy increased from 65.00% (26/40) after initial extraction to 72.50% (29/40) after Self-Refine. On the 40 corresponding perturbed passages, the correct perturbation-following rate increased from 70.00% (28/40) to 72.50% (29/40). Among the 30 negative passages, the number yielding non-empty outputs decreased from 14 to 8, corresponding to a reduction in the non-empty output rate from 46.67% to 26.67%.

The improvement on the original positive passages indicates that Self-Refine corrected some initially inaccurate target extractions. However, the increase in the correct perturbation-following rate was small, suggesting that its ability to respond consistently to controlled changes in the input remained limited. The 20.00-percentage-point reduction in the non-empty output rate indicates that Self-Refine reduced unsupported extraction from passages without eligible targets, although eight negative passages still yielded extracted triples. Because the negative passages were purposively selected from a small sample, these results should be interpreted as a targeted diagnostic assessment rather than an estimate of the false-positive rate across the full corpus.

#### 3.3.3. Remaining Error Analysis

To identify the limitations that remained after refinement and further explain the limited improvement in recall observed in Table 4, the errors in the Self-Refine outputs were manually analyzed based on the subject–predicate–object structure. The errors were classified into four task-specific categories: missing triples, predicate errors, object errors, and invalid triples. A missing triple was a reference triple for which no corresponding prediction was found. A predicate error was a prediction that could be aligned with a reference fact and had the correct subject and object but contained an incorrect or incomplete indicator or factor expression. An object error was an aligned prediction that had the correct subject and predicate but contained an incorrect or incomplete numerical expression, including the value, constraint symbol, unit, or interval boundary. An invalid triple was a prediction that fell outside the task-defined extraction scope, lacked direct textual support, or could not be clearly aligned with any reference triple. After normalization and duplicate removal, each error instance was manually assigned to only one category.

**Figure 5:**
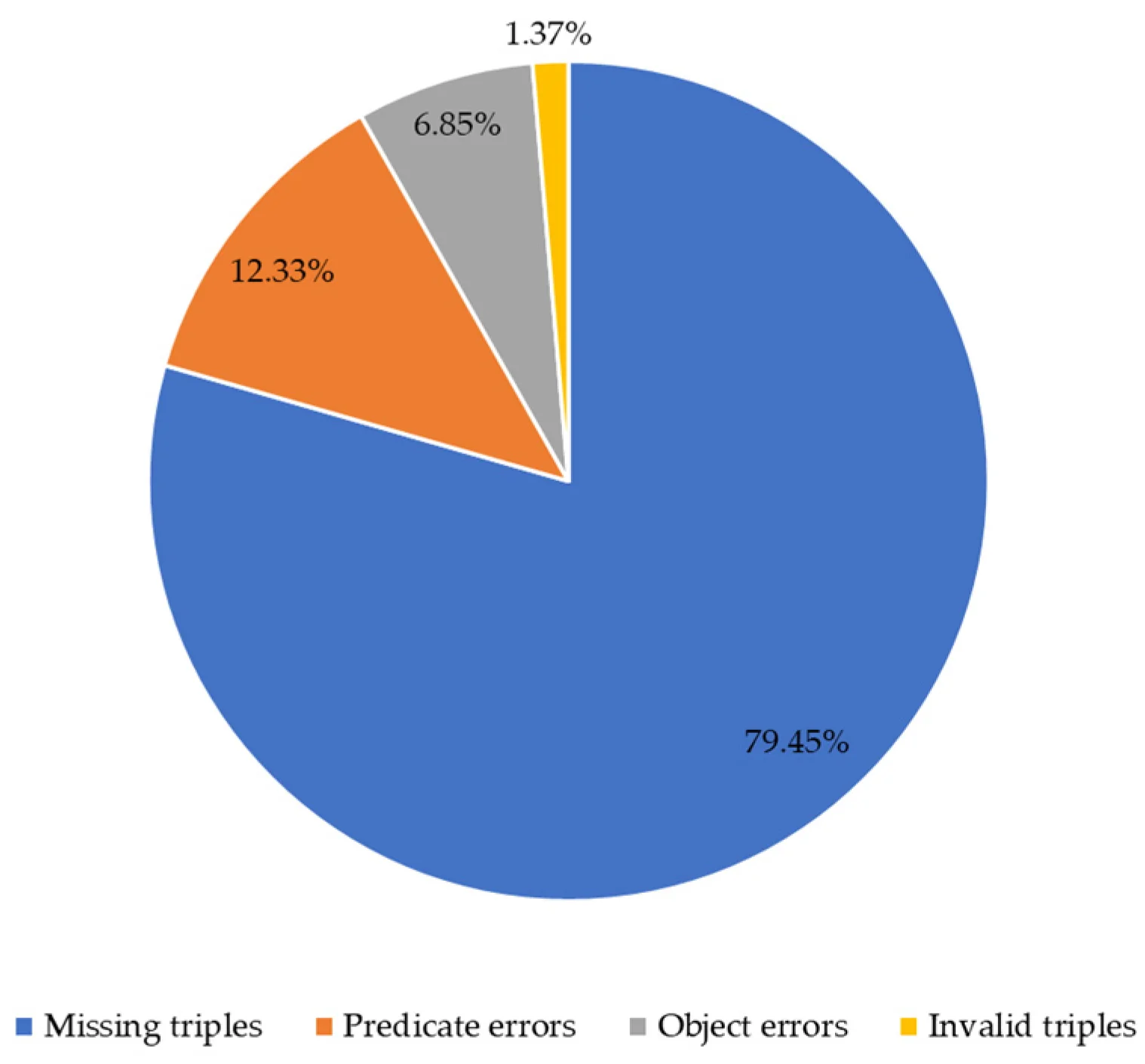
Distribution of remaining error types after Self-Refine.

For example, for the sentence “The limit of cadmium in scallops is ≤2.0 mg/kg,” failure to generate the reference triple “(scallops, cadmium, ≤2.0 mg/kg)” was classified as a missing triple. For “The limit of petroleum hydrocarbons in shellfish is ≤15 mg/kg,” the prediction “(shellfish, polychlorinated biphenyls, ≤15 mg/kg)” was classified as a predicate error because “petroleum hydrocarbons” was incorrectly extracted as “polychlorinated biphenyls.” For “The current national limit for Pb in oysters is ≤1.5 mg/kg,” the prediction “(oysters, Pb, ≤15 mg/kg)” was classified as an object error because the numerical value “1.5” was incorrectly extracted as “15.” Finally, for the sentence “The lead content in cereal-based complementary foods for infants and young children shall be ≤0.2 mg/kg and shall be determined according to GB 5009.12 [25],” the prediction “(cereal-based complementary foods for infants and young children, detection method, GB 5009.12)” was classified as an invalid triple because a detection method does not constitute quantitative food safety information under the task definition. The resulting distribution is presented in Figure 5.

As shown in Figure 5, missing triples constituted the dominant remaining error after Self-Refine, whereas predicate errors, object errors, and invalid triples accounted for smaller proportions. Predicate errors and object errors accounted for 12.33% and 6.85% of the remaining errors, respectively. Predicate errors may associate a numerical limit with an incorrect indicator or hazard factor, while object errors may distort numerical values, units, constraint symbols, or ranges. These errors may affect the interpretation of regulatory requirements and the comparison of detection results with prescribed limits. The predominance of missing triples is consistent with the limited change in recall shown in Table 4. The paired analysis indicated consistent improvements in precision and F1 across the four runs, whereas the 95% confidence intervals for the mean paired recall differences included zero under both matching criteria.

Recovering a completely omitted fact requires the model to re-examine the source text, identify the food object, indicator or hazard factor, and numerical expression, and construct a new triple. This process requires identifying and associating all three fields and may be more demanding than correcting an existing candidate triple, offering a possible explanation for the limited recall improvement. The results suggest that, within the present evaluation, Self-Refine was more effective at increasing the proportion of correctly matched predictions than at recovering reference triples missed during initial extraction. Incomplete information coverage therefore remained a limitation despite the improvements in precision and F1.

### 3.4. Sensitivity Analysis of Similarity-Based Matching

Table 6 presents the F1 scores obtained by re-evaluating the retained outputs under different overall similarity thresholds and field-weight configurations, allowing the observed Self-Refine improvement to be examined beyond the original evaluation configuration.

Under the original weights, F1 ranged from 0.8160 to 0.8220 without Self-Refine and from 0.8368 to 0.8428 with Self-Refine. The F1 improvement remained approximately 0.0208 across all five thresholds. Equal weighting produced the same results in most settings, with a maximum F1 difference of approximately 0.0015 relative to the original weights at a threshold of 0.80. Although absolute F1 decreased slightly at the stricter threshold of 0.95, the direction and magnitude of the Self-Refine improvement remained similar. These results support the consistency of the observed F1 gain within the tested threshold range and weight configurations.

## 4. Conclusions

This study proposes a quantitative food safety information extraction method integrating few-shot prompting and Self-Refine iterative optimization. Without additional model training, the method extracts and associates food objects, indicators or factors, and corresponding numerical expressions. Among the three LLMs, Qwen-Plus achieved the highest initial F1 under both matching criteria. Self-Refine increased strict-match F1 from 0.8146 to 0.8353 and similarity-match F1 from 0.8190 to 0.8398. The run-level paired analysis supported consistent improvements in precision and F1 across the four runs, whereas the evidence for a consistent recall improvement remained limited. Qwen-Plus with Self-Refine also achieved higher F1 than the rule-based baseline under both matching criteria. Furthermore, the F1 improvement over its initial outputs remained positive across the tested overall similarity thresholds and field-weight configurations.

In terms of practical value, the structured extraction results may support the construction of a unified and searchable database of quantitative food safety information for digital document management, knowledge graph construction, and risk parameter management. Such a database could facilitate access by food enterprises to relevant standard provisions and numerical requirements. However, the current schema does not automatically reconstruct implicit exceptions, ambiguous cross-clause applicability, or hierarchical inheritance that is not explicitly supported by the input text or table structure. Therefore, the use of extracted information in decision-support applications requires human verification against the source documents, particularly in these cases. The current corpus comprises Chinese national food safety standards and Chinese-language scientific literature, and the evaluation was conducted on relatively small document samples. The supplementary document-level analysis quantified document-sampling uncertainty on the independent test set and provided additional context for interpreting the observed performance. Future studies should evaluate the framework on larger and more diverse corpora, including international standards, to obtain more precise performance estimates and further examine its generalizability. Future work will extend the stratified evaluation to larger samples and additional knowledge types to further characterize the framework’s performance across different extraction scenarios. In addition, the consistency of the manual error classification remains to be verified. Future studies will use independent classification by multiple evaluators and agreement analysis to strengthen the reliability of the error-distribution analysis.

## Figures and Tables

**Table 1:** Sources and composition of the experimental dataset.

Data Source	Number of Documents	Main Content	Role	Percentage (%)
National food safety standards	149	Normative knowledge, including limit requirements, applicable scopes, and management measures	Main experimental corpus	63.68
Scientific literature	85	Research-oriented knowledge, including risk factors, risk assessment indicators, and mechanisms of action	Supplementary knowledge types and expression forms	36.32
Total	234	—	Overall experimental dataset	100

**Table 2:** Field descriptions and annotation scale of the evaluation reference set.

Annotation Dimension	Field	Description	Count
Food object	subject	Food category or raw material	552 categories
Indicator/factor	predicate	Contaminant, residue, additive, microorganism, or risk indicator	149 categories
Numerical expression	object	Limit value, threshold, detection result, or risk assessment value	137 categories
Unit	unit	Measurement unit	23 types
Constraint symbol	constraint_symbol	≤, ≥, =, or interval	4 types
Valid triple	subject–predicate–object	Complete annotated triple	1031 triples

**Table 3:** Initial triple extraction performance of the rule-based baseline and LLMs.

Model	Matching Method	Precision	Recall	F1
Rule-based baseline	Strict	0.6005	0.4840	0.5360
Qwen-Plus	Strict	0.8269 ± 0.0032	0.8026 ± 0.0036	0.8146 ± 0.0034
DeepSeek-V4-Pro	Strict	0.7782 ± 0.0024	0.8261 ± 0.0050	0.8015 ± 0.0035
Kimi-k2.5	Strict	0.5628 ± 0.0030	0.5965 ± 0.0066	0.5792 ± 0.0045
Rule-based baseline	Similarity, τ = 0.85	0.8809	0.7100	0.7863
Qwen-Plus	Similarity, τ = 0.85	0.8314 ± 0.0034	0.8070 ± 0.0040	0.8190 ± 0.0037
DeepSeek-V4-Pro	Similarity, τ = 0.85	0.7837 ± 0.0034	0.8320 ± 0.0042	0.8071 ± 0.0035
Kimi-k2.5	Similarity, τ = 0.85	0.6152 ± 0.0066	0.6520 ± 0.0047	0.6331 ± 0.0054

LLM results are reported as mean ± SD across four independent runs. The deterministic rule-based baseline was evaluated once. All results correspond to initial extraction without Self-Refine. τ denotes the overall similarity threshold.

**Table 4:** Self-Refine ablation results and run-level paired differences.

Matching Method	Metric	Without Self- Refine	With Self- Refine	Mean Paired Difference [95% CI]
Strict	Precision	0.8269 ± 0.0032	0.8690 ± 0.0016	+0.0421 [0.0384, 0.0458]
Strict	Recall	0.8026 ± 0.0036	0.8041 ± 0.0025	+0.0015 [−0.0033, 0.0063]
Strict	F1	0.8146 ± 0.0034	0.8353 ± 0.0021	+0.0207 [0.0165, 0.0249]
Similarity, τ = 0.85	Precision	0.8314 ± 0.0034	0.8737 ± 0.0021	+0.0423 [0.0351, 0.0495]
Similarity, τ = 0.85	Recall	0.8070 ± 0.0040	0.8084 ± 0.0030	+0.0015 [−0.0073, 0.0102]
Similarity, τ = 0.85	F1	0.8190 ± 0.0037	0.8398 ± 0.0026	+0.0208 [0.0128, 0.0288]

Performance is reported as mean ± SD across four runs. Paired differences were calculated from unrounded per-run metrics as with Self-Refine minus without Self-Refine. Consequently, a reported difference may differ slightly from subtraction of the rounded means. Approximate 95% CIs were calculated using the t distribution with three degrees of freedom and reflect run-level variation on the fixed evaluation set.

**Table 5:** Extraction performance on the 10-document independent test set.

Matching Method	Method	Precision (%)	Recall (%)	F1 (%)
Strict	Without Self-Refine	71.07	82.74	76.46
Strict	With Self-Refine	76.09	86.69	81.05
Similarity, τ = 0.85	Without Self-Refine	76.61	89.19	82.42
Similarity, τ = 0.85	With Self-Refine	80.84	92.10	86.10

**Table 6:** Sensitivity of F1 scores to the overall similarity threshold and field weights.

Overall Threshold	Original Weights, Without Self-Refine	Original Weights, with Self-Refine	Equal Weights, Without Self-Refine	Equal Weights, with Self-Refine
0.75	0.8220	0.8428	0.8220	0.8428
0.80	0.8190	0.8398	0.8205	0.8413
0.85	0.8190	0.8398	0.8190	0.8398
0.90	0.8190	0.8398	0.8190	0.8398
0.95	0.8160	0.8368	0.8160	0.8368

Original weights: ($w_{s}$ = 0.35), ($w_{p}$ = 0.25), and ($w_{o}$ = 0.40); equal weights: ($w_{s}=w_{p}=w_{o}$ = 1/3). The minimum predicate-similarity threshold was fixed at 0.75. Values are F1 scores obtained by re-evaluating the retained model outputs under each configuration.

## Data Availability

Data are available from the corresponding author upon reasonable request.

## Appendix A

### Appendix A.1. Exploratory Comparison with Majority Voting

The 40 source-text units were selected from an eligible pool spanning 16 documents within the existing corpus using document-balanced stratified random sampling. The candidate source-text units were divided into four strata according to document source and content format: national-standard tables, national-standard prose, scientific-literature tables, and scientific-literature prose, with 10 source-text units sampled from each stratum. Selection was based only on document source and content format, without reference to model predictions or reference labels; selected source-text units containing no target relations were not subsequently excluded.

Three settings were evaluated on the same subset: single-pass extraction, majority voting over four independent initial-extraction outputs, and Self-Refine with up to three refinement rounds. Majority voting retained triples appearing in at least three of the four outputs. Self-Refine used the same early-termination criteria as the main experiment: refinement terminated when the feedback indicated that no further revision was required or when the normalized triple set remained unchanged.

**Table A1:** Performance of single-pass extraction, majority voting, and Self-Refine on the 40-unit subset.

Method	Strict Precision (%)	Strict Recall (%)	Strict F1 (%)	Similarity Precision (%)	Similarity Recall (%)	Similarity F1 (%)
Single-pass extraction	84.13	86.30	85.20	89.67	91.99	90.82
Majority voting (≥3/4)	82.67	86.30	84.45	87.62	91.47	89.51
Self-Refine (up to three rounds)	87.17	86.05	86.61	91.62	90.44	91.03

On this subset, Self-Refine achieved the highest precision and F1 under both matching criteria. Majority voting did not improve precision or F1 over single-pass extraction. Recall remained relatively similar across the three settings, indicating that the observed difference was mainly associated with precision. These results are reported as an exploratory supplementary comparison because the analysis was conducted on a limited subset of 40 source-text units.

### Appendix A.2. Supplementary Stratified Results on the Independent Test Set

Supplementary stratified results for the Self-Refine outputs on the 10-document independent test set are reported under strict matching, using exact agreement across the three evaluated fields as a uniform criterion. Table A2 and Table A3 present precision, recall, and F1 by document source and content format, respectively.

**Table A2:** Performance of Self-Refine outputs by document source under strict matching on the independent test set.

Document Source	Precision (%)	Recall (%)	F1 (%)
National food safety standards	79.78	89.75	84.47
Scientific literature	59.18	71.60	64.80

**Table A3:** Performance of Self-Refine outputs by content format under strict matching on the independent test set.

Content Format	Precision (%)	Recall (%)	F1 (%)
Tables	77.41	90.57	83.47
Prose	58.97	50.00	54.12

### Appendix A.3. Document-Level Bootstrap Analysis on the Independent Test Set

To assess uncertainty associated with document sampling, a document-level stratified bootstrap analysis was conducted for the Self-Refine outputs under strict matching. In each bootstrap replicate, four national food safety standards and six scientific articles were sampled with replacement from their respective document-source strata, preserving the original source composition of the independent test set. Each selected document contributed its complete TP, FP, and FN counts; when a document was selected more than once, its counts were included repeatedly. Precision, recall, and F1 were then recalculated from the aggregated counts. A total of 100,000 bootstrap replicates were generated using a random seed of 20261008, and approximate 95% confidence intervals were defined by the 2.5th and 97.5th percentiles of the resulting bootstrap distributions. The results are presented in Table A4.

**Table A4:** Strict-match performance and approximate document-level 95% confidence intervals for Self-Refine outputs on the independent test set.

Metric	Point Estimate (%)	Approximate 95% CI (%)
Precision	76.09	57.89–81.87
Recall	86.69	71.26–91.21
F1	81.05	63.95–86.26

Point estimates are micro-averaged. CI denotes confidence interval. All values are expressed as percentages.

The bootstrap intervals reflect variation caused by resampling complete documents while retaining the original proportions of standards and scientific articles. The observed interval widths should be interpreted together with the limited number of independent test documents and the stratified results in Table A2 and Table A3. The stratified analysis showed higher extraction performance for national food safety standards than for scientific literature and for tables than for prose. Although document-source composition was fixed during resampling, the selected documents could still differ in content format, number of reference triples, and extraction difficulty. These document-level differences may therefore contribute to variation in the aggregated performance estimates.

## Appendix B

### Code Availability

The core code and supplementary prompt materials used in this study are available at the following GitHub repository: https://github.com/tanlz0/Self-Refine-Enhanced-LLM-Framework.git (accessed on 9 October 2026).
